# Methylxanthine and Flavonoid Contents from Guarana Seeds (*Paullinia cupana*): Comparison of Different Drying Techniques and Effects of UV Radiation

**DOI:** 10.1155/2024/7310510

**Published:** 2024-07-02

**Authors:** André Luiz Sampaio da Silva Junior, Madson Moreira Nascimento, Herick Macedo Santos, Ivon Pinheiro Lôbo, Rosilene Aparecida de Oliveira, Raildo Mota de Jesus

**Affiliations:** ^1^ Laboratório de Pesquisa em Química Analítica Departamento de Ciências Exatas Universidade Estadual de Santa Cruz (UESC), Rodovia Jorge Amado, km 16 45662-900, Ilhéus, Bahia, Brazil; ^2^ Centro Universitário SENAI CIMATEC Av. Orlando Gomes, 1845 - Piatã 41650-010, Salvador, Bahia, Brazil

## Abstract

Guarana seeds are typically processed using one of three drying methods: traditional sun exposure, greenhouse drying, or the *alguidar* oven technique. In our research, we evaluated the contents of methylxanthines and flavan-3-ols in sun- and *alguidar*-dried guarana seeds from Bahia State's Low Sul Identity Territory. Caffeine, theobromine, catechin, and epicatechin were determined by high-performance liquid chromatography with UV-visible detection (HPLC/UV-vis). Statistical tools, including analysis of variance (ANOVA), Tukey's test, and exploratory analysis, were employed to analyze the obtained data. Our findings indicated that the flavan-3-ols content in sun-dried guarana samples was lower compared to those dried using the *alguidar* oven, possibly due to exposure to ultraviolet radiation from solar energy. Conversely, we observed no significant differences (*p* > 0.05) in the average contents of methylxanthines between the two drying methods. Our supplementary experiments involving UV-A and UV-C radiation lamps revealed a decreasing trend in methylxanthines and flavan-3-ols contents with increasing duration of UV radiation exposure.

## 1. Introduction

Guarana (*Paullinia cupana* Kunth), a member of the Sapindacea family, is a native fruit of the Amazon region renowned for its stimulant properties. Although it is predominantly cultivated in Brazil, guarana can also be found in limited areas of Venezuela and Colombia. Until the 1980s, the state of Amazonas, Brazil, dominated guarana production, accounting for approximately 90% of the total output. However, spurred by the substantial increase in seed exploitation, the cultivation of guarana has been encouraged in other areas [1]. For commercial purposes, the majority of guarana production is acquired by beverage and pharmaceutical/cosmetic companies or exported to European and North American markets. This underscores the global demand for this prized fruit and its derivatives [1, 2].

The guarana seed is a vital asset in promoting wellness and health revitalization, boasting a chemical profile rich in caffeine (2.5% to 6.0%), phenolic compounds, and essential macro- and microelements [3–6]. Moderate consumption of guarana has been associated with several health benefits such as the prevention of cardiovascular and neurodegenerative diseases [7, 8], antioxidant, antimicrobial, and anti-inflammatory activity [9, 10], as well as protection against DNA damage and antitumor action [11, 12]. In addition, recent studies have further highlighted guarana's potential to enhance human cognitive performance [13] and positively affect energy metabolism in animal models, suggesting promising avenues for addressing obesity-related conditions [14].

The beneficial properties of guarana are related to the high content of natural bioactive compounds, including methylxanthines and flavan-3-ols. Methylxanthines such as caffeine, theobromine, and theophylline, belonging to the purine alkaloid group, are methylated derivatives of xanthine (2,6-dihydroxypurine). These compounds (especially caffeine) antagonize adenosine receptors and exert stimulant effects on the central nervous system, reducing fatigue and increasing alertness or reaction time [13]. Besides guarana, other typical sources of caffeine include coffee and tea. Flavan-3-ols such as catechin and epicatechin are polyphenols found in several plants and fruits. Characterized by a basic structure comprising two aromatic rings and a dihydropyran heterocycle with a hydroxyl group at carbon 3 [15], catechin and epicatechin are renowned for their potent antioxidant properties. The main mechanism of action involves the scavenging of free radicals by the multiple hydroxyl groups on the aromatic rings, thereby potentially preventing age-related diseases (e.g., Parkinson's and Alzheimer's disease). Previous studies reviewing the effects of guarana on human health have reported that processing steps may affect the seed's bioactive profile [1, 14, 16]. Thus, it is imperative to assess the content of methylxanthines and flavan-3-ols in guarana subjected to various postharvest treatments. Such evaluations are pivotal for ensuring the maintenance of guarana's beneficial properties and its continued contribution to human health and well-being.

The most critical postharvest step for ensuring guarana quality is the drying technique. According to Brazilian legislation, the maximum moisture content for the commercialization of guarana seeds should be up to 12% [17]. As with the drying of cocoa and coffee beans, further parameters are also relevant to evaluate the final quality of the product, including drying temperature, sun exposure, roasting effects, losses by volatilization or degradation, oxidation process, and studies involving fermentation [18–23]. However, few studies are focusing on specific methodologies for guarana seed processing. Due to a lack of standardization in the guarana production chain, different techniques are employed for the seeds' drying. In this sense, factors such as high UV radiation exposure time, fungal degradation from poor moisture control, and exposure to high temperatures can contribute to the degradation of bioactive compounds and affect the final quality of the dried seeds.

UV radiation can increase the biochemical activity in foods, reduce the formation of fungi, and contribute to higher development in plant roots [24–26]. However, this radiation also causes the loss of volatile compounds and the degradation of fatty acids and phenolics, as well as an incomplete drying process that results in high moisture content. Several studies indicated that UV radiation can lead to the production of high concentrations of aromatized carbonyls, consequently impacting the flavor profile of products like cocoa and coffee [27–29].

In Bahia State's Low Sul Identity Territory (Northeastern Brazil), three different techniques for drying the guarana seeds have been used: sun exposure, greenhouse, and *alguidar* oven. Sun-drying is the most common technique, in which the guarana seeds are placed under a black tarp and exposed to solar energy for 5–7 days. In greenhouse drying, solar energy is also employed for seed drying through natural convection, typically lasting around 3 to 5 days. However, a closed structure is assembled to protect the seeds from external contamination. Although both techniques use natural energy, sun- and greenhouse-drying expose the guarana seeds to intense solar radiation and require a longer time for efficient drying. On the other hand, the *alguidar* oven is a drying technique that does not depend on weather conditions. The guarana seeds are dried for only 2–4 hours using an iron plate heated by thermal energy from the biomass burning (see Supplementary Figure 1). However, the high temperatures and smoke generated during the process are the primary drawbacks of alguidar drying. Some of these factors can adversely affect the chemical composition and quality of the guarana seeds, resulting in irreversible impacts on the final product.

The current study is aimed at assessing the levels of caffeine, theobromine, catechin, and epicatechin in guarana seed samples dried using both sun and *alguidar* methods, sourced from the Low Sul Identity Territory of Bahia State. In addition, further experiments using UV-A and UV-C lamps under controlled conditions were performed to evaluate the effects of UV radiation on the contents of bioactive compounds in guarana.

## 2. Material and Methods

### 2.1. Reagents, Solutions, and Samples

Organic solvents such as 2-propanol and methanol (chromatographic grade) were acquired from Sigma-Aldrich (St. Louis, MO, USA). Analytical standards of caffeine (1,3,7-trimethylxanthine), theobromine (3,7-dimethylxanthine), (+)-catechin, and (-)-epicatechin were purchased from Sigma-Aldrich (St. Louis, MO, USA). Stock solutions of caffeine, theobromine, catechin, and epicatechin (1000 mg L^−1^) were prepared using ultrapure water of resistivity 18.2 M*Ω* (Milli-Q, Merck KGaA, Darmstadt, Germany), filtered using a Millex-HV 0.45 *μ*m PVDF filter (Merck, Darmstadt, Germany), and stored at -20°C until analysis.

Twenty-eight guarana samples dried using two different drying techniques (conventional sun exposure and *alguidar* oven) were collected from producer cities located in Bahia State's Low Sul Identity Territory: seven samples from Camamu (-13°56′25.5^″^, -39°9′43.0^″^), five samples from Taperoá (-13°37′54.22^″^, -39°11′55.61^″^), eight samples from Nilo Peçanha (-13°38′04^″^, -39°06′45^″^), and eight samples from Valença (-13°19′52.00^″^, -39°16′0.28^″^). Sun-dried samples (*n* = 14) were subjected to solar exposure on a polyethylene tarpaulin for seven days, whereas *alguidar*-dried samples (*n* = 14) were placed on an iron plate furnace and heated for 2–3 hours. Both drying techniques were performed by the producers themselves.

Additionally, samples of guarana seeds *in natura* (*Paullinia cupana* var. *sorbilis*) were obtained from ripe fruits for the experiments involving UV radiation. The samples were harvested following the Brazilian Agricultural Research Corporation (EMBRAPA). Guaranas from healthy plants (free of spots or insects) producing at least one kilogram of seeds per year were selected. After pulping, guarana seeds were mixed, homogenized, and freeze-dried. Then, all samples were ground using a model A11 basic analytical mill (IKA, São Paulo, Brazil). Freeze-dried and grounded seeds were used as control samples (i.e., not exposed to UV radiation) in experiments using UV lamps.

### 2.2. Equipment

UV-C (9 W) lamps at wavelengths 254 and 252 nm and UV-A (9 W) at 350 nm were used for experiments with UV radiation. A model CR/100 electronic oven (Sterilifer, São Caetano do Sul, Brazil) equipped with digital temperature control was used for drying the guarana seeds with high moisture content. A model FreeZone freeze dryer (Labconco, Kansas City, MO, USA) was used for sample lyophilization. The moisture content was determined using a model G650i portable grain moisture meter (Gehaka, São Paulo, Brazil). For the extraction procedure and mobile phase degassing, a model UP30H Elmasonic ultrasonic bath (Elma, Frechen, Germany) was used. The mobile phase was filtrated using a model ME 1C vacuum pump (Vacuubrand, Wertheim, Germany).

A model Prominence LC-20A HPLC Shimadzu (Kyoto, Japan) equipped with a UV-vis detector set at a wavelength of 274 nm and a C18 column Kinetex^®^ (100 mm length, 2.6 *μ*m particle size, and 100 Å pore size) from Phenomenex (Torrance, CA, USA) were used for chromatographic analysis. The separation conditions were established according to Nascimento et al. [30].

### 2.3. Analytical Procedures for Extraction of Methylxanthines and Flavan-3-Ols

Caffeine, theobromine, catechin, and epicatechin were extracted from guarana samples according to procedures described by Nascimento et al. [30]. For methylxanthines, a sample mass of 50 mg was added to 10.0 mL of 0.2 mol L^−1^ NaOH and 60 mL of ethyl acetate. After slow stirring for 10 min, the organic fraction was collected in a 500 mL flask, and the aqueous phase was twofold reextracted using 40 mL of ethyl acetate. Finally, the ethyl acetate present in the organic phase was evaporated at 60°C, and the dried extracts were suspended in 10 mL of ultrapure water. For flavan-3-ols, 10 mL of a mixture of water/acetone (1/1) was added to 100 mg of sample mass. The system was stirred in a vortex for 1 min and subjected to ultrasound-assisted extraction for 5 min at 45°C and 50% of maximum power. The extracts were adjusted to 10 mL using ultrapure water. After extraction procedures, the samples were 100-fold diluted and filtered using a 0.45 *μ*m Millex filter (Merck, Darmstadt, Germany) before injection into HPLC. All extractions were performed in triplicate for each sample.

### 2.4. Exposure of Guarana Seeds to UV Radiation

To study the influence of UV radiation on the contents of bioactive compounds in guarana seeds, two lamps of different wavelengths were chosen to evaluate the amplitude extremes of the UV radiation range, i.e., UV-A (350 nm) and UV-C (252; 254 nm). The experiments were performed using a black wooden box, whose cover had an open-close mechanism for removing the samples and preventing the incidence of external radiation. A sample mass of approximately 100 g of guarana seeds was exposed to UV radiation for 0 (control sample), 6, 12, 24, 36, 48, and 60 hours.

### 2.5. Data Processing

The results for methylxanthine and flavan-3-ols contents in guarana seeds subjected to different drying conditions were evaluated by analysis of variance (ANOVA). To assess whether the contents of the bioactive compounds from guarana seeds in control (freeze-dried and nonirradiated) and UV-treated samples differ significantly, the statistical Tukey test at the 95% confidence level was applied. Chemometric tools such as principal component analysis (PCA) and hierarchical cluster analysis (HCA) were used to check the correlation between variables and clustering trends between samples. All statistical/chemometric analyses were performed using Statistica software 8.0 (Stat-Soft, USA). Microsoft Excel 2010 (Microsoft, USA) was used for mathematical operations.

## 3. Results and Discussion

### 3.1. Methylxanthine and Flavan-3-Ols Content in Sun- and *Alguidar*-Dried Guarana Seeds

**Figure 1:**
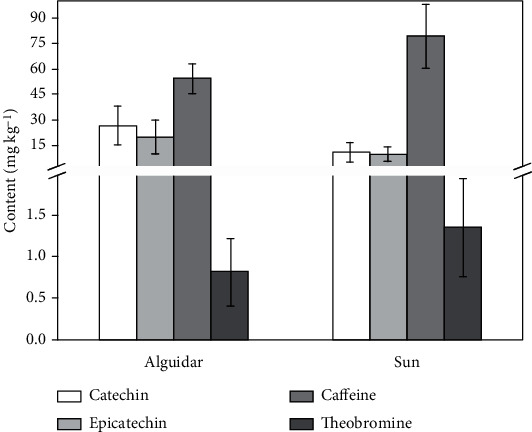
The average content of methylxanthines and flavan-3-ols in guarana samples subjected to different drying techniques.

The *alguidar* oven presented the best results for the drying efficiency of guarana seeds, providing a low moisture content (about 8%). In turn, moisture contents ranging from 10 to 16% were obtained using the sun-drying technique. To prevent the action of fungi and follow the guidelines of Brazilian legislation [17], the moisture of the sun-dried guarana samples was adjusted to approximately 8% by drying in an electronic oven before grinding.  Figure 1 shows the average contents of methylxanthines and flavan-3-ols in guarana samples.

Similar contents of catechin and epicatechin were found for *alguidar*-dried seeds (20–26 mg kg^−1^). As expected, caffeine was the major compound in guarana.  Figure 1 shows that the average contents of caffeine and theobromine in seeds subjected to *alguidar* drying were 54 and 0.8 mg kg^−1^, respectively. For sun-dried seeds, the average content of flavan-3-ols was around 10 mg kg^−1^, whereas a value of approximately 1.3 mg kg^−1^ was found for theobromine. Slightly higher caffeine contents were found for sun-dried guarana (>75 mg kg^−1^). A statistical comparison using ANOVA (*p* < 0.05) showed a significant difference in the average contents of catechin and epicatechin in guarana samples dried by the different techniques (Supplementary Tables 1 and 2). No statistical difference was observed for caffeine and theobromine in sun- or *alguidar*-dried samples. Similar trends were previously reported for caffeine content in coffee beans subjected to heat-pump- and sun-drying [29] or hot-air- and sun-drying [31]. Different drying techniques do not affect methylxanthine contents, probably due to their high thermal stability.

An exploratory analysis using PCA was applied to check the correlation between variables and samples. For this purpose, a data matrix consisting of 28 samples arranged in rows and 4 variables (catechin, epicatechin, theobromine, and caffeine) in columns was obtained. The dataset was preprocessed by the autoscaling method to provide the same degree of importance for all variables [32]. The criterion for PC extraction was based on a scree test and eigenvalues ≥ 1.

Table 1 shows that three principal components (PCs) were extracted according to the established criteria. The first principal component (PC1) explained 54.1% of the total variance, where the variables catechin and epicatechin presented high loadings. Thus, PC1 was characterized by the discrimination of samples according to the content of phenolic compounds. PC2 explained 35.9% of the variance and was characterized by high theobromine loading. The variable caffeine presented a high loading in PC3.

**Figure 2:**
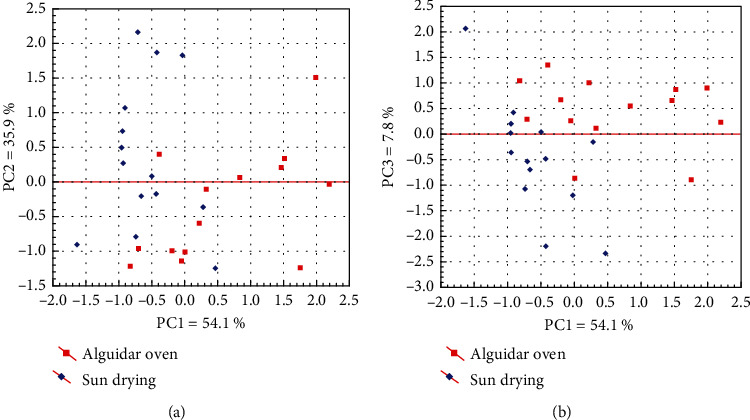
Score plots generated from the principal component analysis. (a) PC1 vs. PC2; (b) PC1 vs. PC3. Sun- and *alguidar*-dried samples are highlighted in red and blue points, respectively.

As seen in score plots (Figure 2), a group of guarana dried by *alguidar* oven was discriminated from the sun-dried samples in PC1 based on the high content of catechin and epicatechin. On the other hand, most sun-dried samples were separated with negative loadings in PC1, indicating a low content of phenolic compounds. In PC2 and PC3, a trend for the separation of sun-dried samples was observed, probably due to the high content of theobromine and caffeine. The greater stability of methylxanthines against photolysis (compared to flavonoids) may explain the higher contents of caffeine and theobromine in guarana seeds subjected to the sun-drying technique [33].

**Figure 3:**
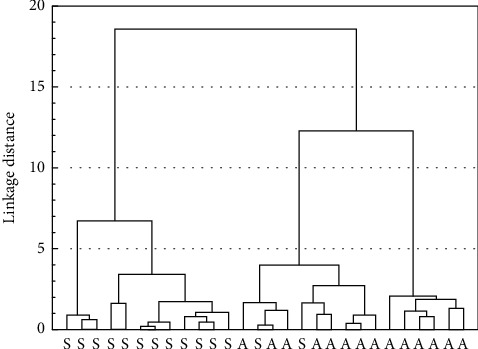
Hierarchical cluster analysis of guarana samples dried by two different techniques. L distance on the *y*-axis. S: sun-drying; A: *alguidar*-drying, on the *x*-axis.

A hierarchical cluster analysis (HCA) was performed to identify possible similarities between the guarana samples and confirm the trends observed from the PCA results. For this purpose, a dendrogram containing the rows (samples) was obtained using the Ward method and the Euclidean distance. As shown in Figure 3, two clusters were formed according to the drying technique. One cluster consisted exclusively of sun-dried samples (S), and the other consisted mostly of samples dried in an *alguidar* oven (A). Two samples “S” were clustered with the *alguidar*-dried samples in the second group, probably due to the high content of catechin and epicatechin. Previous studies reported that guarana seeds cultivated in the same areas present similar chemical compositions and show a clustering trend from PCA analysis [34, 35]. Our results suggest that the drying technique also appears to influence the contents of flavan-3-ols and methylxanthines in guarana seeds.

### 3.2. Influence of UV Radiation on the Content of Bioactive Compounds in Guarana Seeds

#### 3.2.1. Effects on Flavan-3-Ols

We expected lower contents of phenolic compounds in guarana samples dried in an *alguidar* oven. In this technique, the drying occurs on a heated iron plate (temperatures of up to 250°C), which can act by accelerating oxidation reactions, mainly for phenolics. However, the sun-dried samples presented lower contents of catechin and epicatechin (see Figure 1 and Supplementary Table 1). Thus, the incidence of solar radiation (UV) for a prolonged time appears to be the predominant factor in the degradation of flavan-3-ols. Such results corroborate those reported by Scattino et al. [36], who observed a decrease in catechin content in a peach cultivar after postharvest UV irradiation.

**Figure 4:**
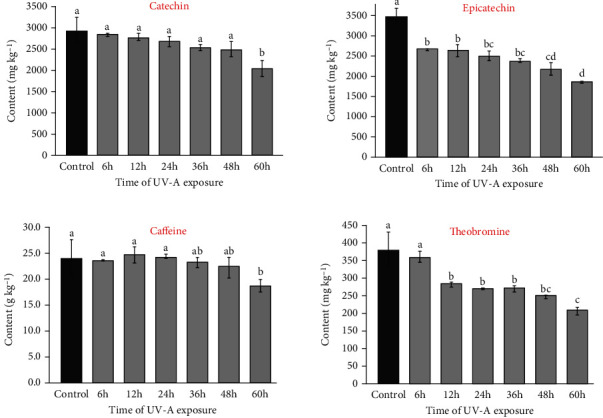
Methylxanthines and flavan-3-ols content in guarana samples subjected to different UV-A exposure times. Means followed by different lowercase letters indicate significant differences by the Tukey test (*p* < 0.05).

**Figure 5:**
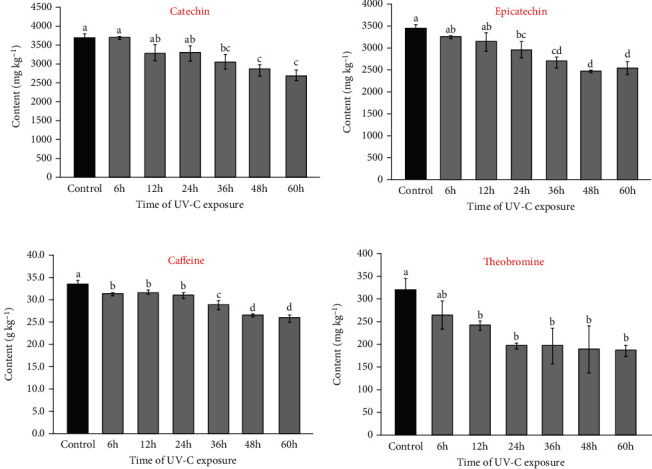
Methylxanthines and flavan-3-ols content in guarana samples subjected to different UV-C exposure times. Means followed by different lowercase letters indicate significant differences by the Tukey test (*p* < 0.05).

Controlled experiments using UV-A and UV-C lamps were performed to assess the effects of radiation on the content of bioactive compounds in guarana seeds (Figures 4 and 5). Preliminary tests using UV-B lamps were also performed. However, as poor precision results (RSD > 40%) were obtained for methylxanthines and flavan-3-ols after HPLC analysis (data not shown), these experiments will not be discussed in this study. The Tukey test showed a significant difference in epicatechin content in guarana seeds after 6 hours of UV-A radiation exposure, whereas catechin content significantly decreased after 60 hours of UV-A incidence (Figure 4). Approximately 33% and 26% degradation percentages were observed for epicatechin and catechin, respectively, after 60 hours of UV-A exposure.

The significant loss of epicatechin (reduction from 3,497 to 2,331 mg kg^−1^ after 60 h) was carefully evaluated in this experiment. Considering that both compounds (catechin and epicatechin) have similar structures, we expected similar degradation kinetics. However, a lower degradation was found for catechin (from 2,927 to 2,039 mg kg^−1^), which may be related to the higher steric impediment of the *trans*-isomer. Flavonoids have ideal structures for capturing free radicals and act as antioxidants, whose activity as “H” and electron donor agent is proportional to the number of hydroxyls. Such compounds have also been widely studied for cancer prevention due to their action against these radicals [1, 4, 37]. Gadkari and Balaraman [33] reported that the extraction and identification of catechins are critical steps, as these compounds can be commonly linked to sugar and proteins or interact with several chemical structures present in the matrix. Our UV experiments demonstrated that catechins are sensitive to light-induced degradation, underscoring the need for careful handling to preserve their quality in products.

Catechin and epicatechin can undergo epimerization when exposed to UV radiation. Kothe et al. [38] observed similar trends in cocoa beans subjected to roasting, where epicatechin content decreased notably in two examined batches while catechin content increased. The authors also reported that other variables can act by accelerating the mechanism, and an in-depth study is needed. Therefore, these results agree with those presented in our study.

In UV-C experiments, the Tukey test showed a statistical difference at a 95% confidence level for catechin content in guarana seeds after 36 hours of exposure. As seen in Figure 5, epicatechin content significantly decreased after 24 hours of UV-C incidence. After 60 hours of treatment, losses of around 27% were found for both catechin and epicatechin. Similar to methylxanthines, where the UV absorption range is 210–290 nm, the chemical bonds in flavonoids can similarly break down, leading to the formation of molecule fragments or free radicals within the matrix and potentially undergoing epimerization [39, 40].

#### 3.2.2. Effects on Methylxanthines

The effects of UV radiation on the methylxanthines were also studied. As seen in Figure 4, UV-A radiation decreased the caffeine and theobromine content depending on exposure time. Compared to control samples (i.e., freeze-dried/nonirradiated), a decrease of approximately 22% (from 24.0 to 18.6 g kg^−1^) and 45% (from 382 to 207 mg kg^−1^) was found for the caffeine and theobromine content, respectively, after 60 h of UV-A exposure. The Tukey test showed that the average content of caffeine was significantly affected by 60 h of exposure, whereas theobromine content suffered significant degradation from 12 h of UV-A incidence.

Losses of caffeine and theobromine after 60 hours of UV-A exposure indicate a significant trend of photochemical degradation. This process is attributed to oxidative stress induced by UV-A irradiance (350 nm), leading to the formation of free radicals through the homolysis of residual water or other compounds in the matrix, resulting in the breaking of chemical bonds and the generation of radical species [41, 42]. Guarana composition provides a wide range of compounds with UV-vis absorption (210–800 nm). Some of these compounds can be degraded due to a broken chemical bond or suffer radical breaking catalyzed by UV light, acting as free radicals. Due to their instability, these radicals may attack and promote the degradation of caffeine and theobromine [37, 43].

The incidence of UV-C radiation on guarana seeds also affected the methylxanthine contents. Compared to control samples, caffeine content decreased from 33.7 to 25.9 g kg^−1^ (23%) after 60 h of UV-C exposure. Theobromine content decreased by 42%, ranging from 325 to 187 mg kg^−1^. According to the Tukey test, a significant difference at a 95% confidence level was observed for caffeine and theobromine contents after 6 h and 12 h of UV-C exposure, respectively. In this case, there is a possibility of direct interaction between the compounds and UV-C radiation since the absorption range of caffeine and theobromine is 210 and 290 nm [1, 44]. This strong UV radiation interaction may result in the breaking of the aromatic ring bonds of the molecules, leaving only molecular compound fragments.

## 4. Conclusion

Guarana samples from Bahia State's Low Sul Identity Territory were analyzed for their methylxanthines and flavan-3-ols content. The exploratory data analysis using PCA and HCA enabled us to distinguish between guarana seeds based on the drying method. Guarana seeds dried through conventional sun exposure exhibited slightly higher caffeine and theobromine contents. On the other hand, sun-dried guarana presented undesirable moisture contents (>10%) and lower contents of catechin and epicatechin compared to samples dried in an *alguidar* oven. The experiments using UV lamps showed that radiation at UV-A and UV-C wavelengths decreased the contents of methylxanthines and flavan-3-ols in guarana seeds. This study provides novel insights into the composition of methylxanthines in guarana seeds subjected to various drying techniques. It suggests that prolonged exposure to sunlight (>2.5 days) could lead to a reduction in the bioactive compound content in guarana. Our finding also suggests that the flavan-3-ols content could be a valuable biomarker for assessing guarana quality. Although seeds dried using the *alguidar* method exhibited higher levels of catechin and epicatechin, further investigations are necessary to evaluate their sensory properties and the potential production of polycyclic aromatic hydrocarbons.

## Figures and Tables

**Table 1:** VARIMAX rotated factor loadings.

Variables	PC1	PC2	PC3
Catechin	**0.9695**	-0.1432	0.0634
Epicatechin	**0.9466**	0.1315	0.2088
Caffeine	-0.1899	0.3954	**-0.8959**
Theobromine	0.0088	**0.9333**	-0.3480
Total variance (%)	54.1	35.9	7.8
Cumulative (%)	54.0	89.9	97.7

The highest loading values are highlighted in bold.

## Data Availability

All data generated or analyzed during this study are included in this manuscript and its supplementary information files.

## Supplementary Materials

Supplementary Materials Supplementary Figure 1: (A) model of an *alguidar* oven. (B) Procedure for drying of guarana seeds using *alguidar*. Supplementary Table 1: catechin, epicatechin, caffeine, and theobromine contents in guarana samples subjected to *alguidar*-drying (*n* = 14) and sun-drying (*n* = 14) techniques. Supplementary Table 2: ANOVA results for methylxanthines and flavan-3-ols content in guarana samples subjected to *alguidar*- and sun-drying techniques.
