# Supplementary material for: Methylxanthine and Flavonoid Contents from Guarana Seeds (Paullinia cupana): Comparison of Different Drying Techniques and Effects of UV Radiation
Source: Int J Food Sci. 2024 Jul 2;2024:7310510. doi: 10.1155/2024/7310510 (PMC11233186; doi:10.1155/2024/7310510)
Supplement: Supplementary Materials — Supplementary Figure 1: (A) model of an alguidar oven. (B) Procedure for drying of guarana seeds using alguidar. Supplementary Table 1: catechin, epicatechin, caffeine, and theobromine contents in guarana samples subjected to alguidar-drying (n = 14) and sun-drying (n = 14) techniques. Supplementary Table 2: ANOVA results for methylxanthines and flavan-3-ols content in guarana samples subjected to alguidar- and sun-drying techniques. [file 7310510.f1.pdf]

Methylxanthines and flavonoids contents from guarana seeds (*Paullinia cupana*):

comparison of different drying techniques and effects of UV-radiation

<u>Supplementary Material</u>

Supplementary Figure 1. (A) Model of an *alguidar*-oven; (B) Procedure for drying of guarana seeds using *alguidar*.

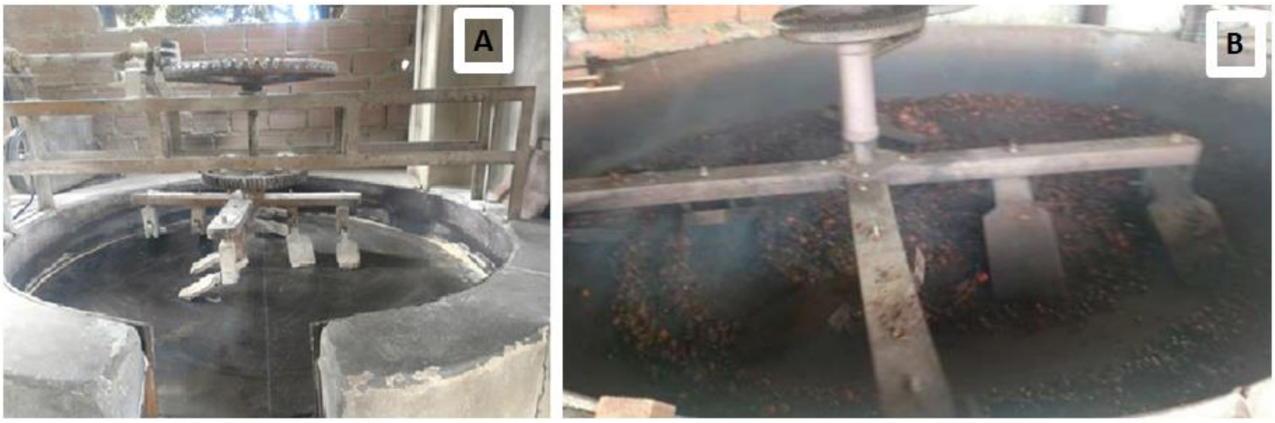

Note: Pictures taken by the authors themselves.

Supplementary Table 1. Catechin, epicatechin, caffeine, and theobromine contents in guarana samples subjected to *alguidar*-drying (n = 14) and sun-drying (n = 14) techniques.

**Bioactive compound**							
**Catechin (mg kg^{-1})**		**Epicatechin (mg kg^{-1})**		**Caffeine (mg kg^{-1})**		**Theobromine (mg kg^{-1})**	
**Drying technique**							
Alguidar	**Sun**	***Alguidar***	**Sun**	***Alguidar***	**Sun**	***Alguidar***	**Sun**
46.39	9.36	32.18	14.27	53.26	71.11	1.08	1.03
25.93	11.26	15.91	8.56	42.75	105.63	0.61	1.40
32.17	8.04	33.46	8.83	51.12	70.45	1.00	1.60
37.27	6.97	27.78	7.36	50.65	78.77	1.10	1.28
37.03	10.14	37.65	8.06	57.23	93.01	1.68	2.45
12.33	7.45	9.01	8.04	57.57	72.24	0.48	1.45
23.29	11.83	11.49	11.87	52.19	90.18	0.46	2.23
21.44	10.59	11.24	8.63	73.16	80.22	0.73	1.09
27.43	17.97	24.47	12.13	55.56	99.25	0.98	2.40
22.25	23.27	18.52	14.45	63.42	96.76	0.99	0.84
13.39	21.36	7.96	17.99	42.21	66.58	0.21	0.88
20.51	5.24	12.24	2.67	47.47	29.41	0.43	0.19
11.73	8.41	17.48	8.30	50.03	83.64	0.95	0.79
43.78	7.75	23.84	7.40	64.34	73.18	0.68	1.35

Supplementary Table 2. ANOVA results for methylxanthines and flavan-3-ols content in guarana samples subjected to *alguidar*- and sun-drying techniques.

**Source of Variation**	**SS**	**df**	**MS**	**F-calc.**	***p*-value**	**F-crit.**
**Catechin**						
Between groups	1222.3	1	1222.3	18.2	0.00027	4.26
Within groups	1612.5	24	67.1			
Total	2834.9	25				
**Epicatechin**						
Between groups	617.9	1	617.9	11.8	0.00214	4.26
Within groups	1254.7	24	52.3			
Total	1872.6	25				
**Caffeine**						
Between groups	4230.5	1	4230.5	18.5	0.00024	4.26
Within groups	5466.3	24	227.7			
Total	9696.9	25				
**Theobromine**						
Between groups	2.26	1	2.27	7.66	0.01071	4.26
Within groups	7.11	24	0.29			
Total	9.38	25				

SS = sum of squares; df = degree of freedom; MS = mean of squares; F-calc. = calculated value for F; F-crit. = critical value for F at 95 % of confidence level.
